# Realization of Multi-Stable Ground States in a Nematic Liquid Crystal by Surface and Electric Field Modification

**DOI:** 10.1038/srep11368

**Published:** 2015-06-23

**Authors:** Jin Seog Gwag, Young-Ki Kim, Chang Hoon Lee, Jae-Hoon Kim

**Affiliations:** 1 Department of Physics, Yeungnam University, Gyeongsan 712-749, Korea; 2 Liquid Crystal Institute and Chemical Physics Interdisciplinary Program, Kent State University, OH 44242, USA; 3 Samsung Electronics, LCD R&D Center, Yongin 446-811, Korea; 4 Department of Electronics and Computer Engineering, Hanyang University, Seoul 133-791, Korea

## Abstract

Owing to the significant price drop of liquid crystal displays (LCDs) and the efforts to save natural resources, LCDs are even replacing paper to display static images such as price tags and advertising boards. Because of a growing market demand on such devices, the LCD that can be of numerous surface alignments of directors as its ground state, the so-called multi-stable LCD, comes into the limelight due to the great potential for low power consumption. However, the multi-stable LCD with industrial feasibility has not yet been successfully performed. In this paper, we propose a simple and novel configuration for the multi-stable LCD. We demonstrate experimentally and theoretically that a battery of stable surface alignments can be achieved by the field-induced surface dragging effect on an aligning layer with a weak surface anchoring. The simplicity and stability of the proposed system suggest that it is suitable for the multi-stable LCDs to display static images with low power consumption and thus opens applications in various fields.

Liquid crystals (LCs) have the preferred orientation of molecules, the so-called director 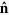, which can be controlled by weak physical cues, such as surface modification and electromagnetic fields^1^. This unique feature of LCs enabled the advent of liquid crystal displays (LCDs). Every LCD device such as TVs, monitors and cell phones has a stable director alignment in the field-free state (ground state). The director alignment in a ground state is the crucial factor to determine the characteristics of LCD and is generally classified into two categories, planar alignment and vertical alignment^2^,^3^. The planar alignment has the ground state in which the director 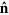 is tangentially orientated (parallel to alignment layers), while in the ground state of a vertical alignment, 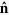 is aligned homeotropically (perpendicular to alignment layers). On the basis of two different alignments as a ground state, LCD technologies have made remarkable development. As LCDs accordingly successfully fulfill the fast-switching, high-resolution, low cost, and thin flat panel displays, they have been evolved into major display devices by replacing other display devices such as a cathode ray tube (CRT) and a plasma display panel (PDP).

Recently, with the significant drop of LCD price and the efforts to save natural resources, LCDs are even replacing paper to display static images such as the menu in restaurants, price tags in stores, and advertising boards on street. In order to meet such a huge market demand on the LCDs for static images, the LCD that is able to have multi-stable ground states, the so-called multi-stable LCD^3^,^4^,^5^, comes into the limelight due to its great potential for low power consumption. If the device is able to set a desired image as its ground state, it would consume much less electricity because it no longer needs to switch directors for displaying an image. In current LCDs, however, it is not trivial to alter the ground state once it is established since the alignment layer having a strong surface anchoring strength has to be adopted for better performance in displaying images.

In this work, we propose a new technique to realize the multi-stable ground states in a single LC cell by taking an advantage of a surface gliding effect on the aligning surface with weak surface anchoring. We experimentally and theoretically demonstrate that when a sufficient electric field is applied for a certain amount of time in the proposed cell, the directors not only in a bulk but also on an aligning surface are reorientated and thus the ground state can be reestablished. In this work, we verified that numerous ground states can be generated with various combinations of applied electric fields, and are stable thermally and electrically.

## Results

**Figure 1:**
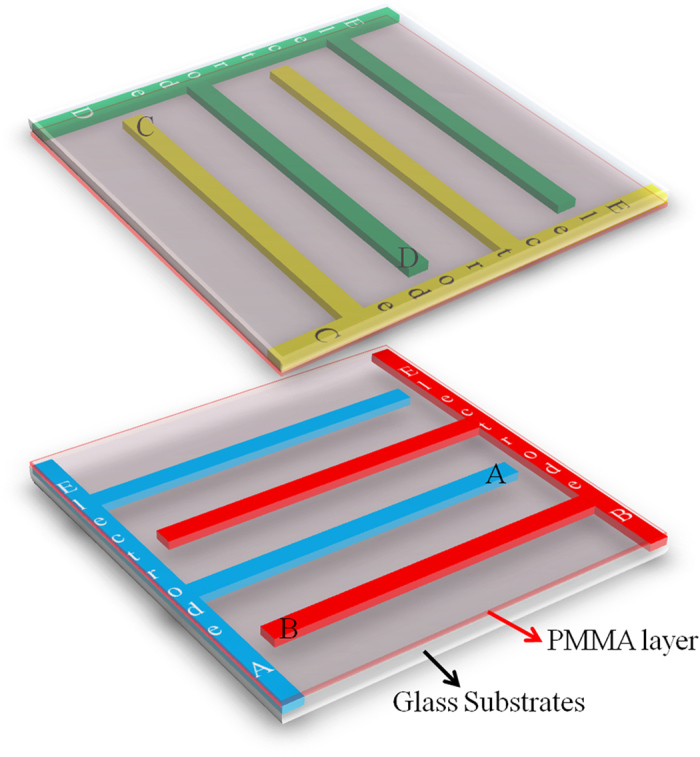
Schematic diagram of the proposed cell for multi-stable ground states. The glass substrates with interdigitated aluminum electrodes are spin-coated with a PMMA and are subsequently assembled in the manner that the electrodes on top and bottom substrates are crossed.

Figure 1 shows the configuration of the proposed LC cell for the multi-stable ground states. The interdigitated electrodes were prepared by sputtering an aluminum on glass substrates; the electrode width is 20 μm and the gap between electrodes is 60 μm. In order to achieve a weak anchoring surface, we used the polymethyl-methacrylate (PMMA) as a tangential aligning layer in which the director 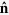 of used LC is aligned parallel to the layer. The glass substrates with aluminum electrodes were spin-coated by PMMA and were then assembled in the manner that the electrodes on a top and a bottom substrates are crossed (Fig. 1). The cell thickness, *d* = 6 μm, was set by glass spacers mixed with UV-glue NOA 65 (Norland Products, INC.) which was also used to seal the cells.

In the experiments, we used the LC, ZKC-5085XX (Merck Korea). The LC is of positive dielectric anisotropy; Δ*ε* > 0. The phase diagram of the LC confirmed by polarizing optical microscopy (POM) upon cooling is Cr (<20 °C) N (120 °C) I where Cr, N, and I are crystal, nematic and isotropic phases, respectively. To prevent any possible surface memory effect^6^,^7^, the LC was injected into the cells above the N-I transition temperature, *T*_NI_(=120 °C). The temperature *T* was controlled by a hot-stage HS82 (Mettler-Toledo International Inc.) and a slow rate of temperature change (1 °C/min) was adopted to minimize the effect of thermal flows^8^,^9^,^10^.

In the LC cells with classical aligning agents such as a polyimide, the directors in a bulk are easily reorientated in response to an electric field *E*. Although a strong field is introduced, however, due to a strong surface anchoring of a polyimide layer, it is difficult to realign the surface directors 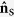 that are the directors in the regions from the aligning layer *z* = 0 to *z* ~ *ξ*_*e*_ where *ξ*_*e*_ is an electric coherence length^1^.

In the proposed cell (Fig. 1), thank to a weak surface anchoring of the PMMA layer, *E* is able to realign the directors not only in the bulk but also in the surface region because *ξ*_*e*_ decreases as the surface anchoring strength *W* becomes weaker. The reorientation of surface directors, the so-called surface gliding, has been studied using a range of techniques^11^,^12^,^13^,^14^,^15^,^16^,^17^,^18^,^19^,^20^,^21^,^22^,^23^,^24^. Furthermore, the relatively low phase transition *T* of PMMA from Cr to a glass (G) phase, 
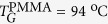
, allows one to further reduce *W* by elevating *T* above 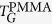. Therefore, at 120 °C( = *T*_NI_) > *T* > 94 °C one is able to readily realign the surface directors 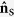 by applying *E* above a critical value E*; E* increases with the decrease of *T*. Subsequently, as the temperature is lowered below 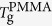, the increased *W* enable the PMMA layer to memorized the reorientated director profile. As a result, a new ground state is established after the field is removed.

**Figure 2:**
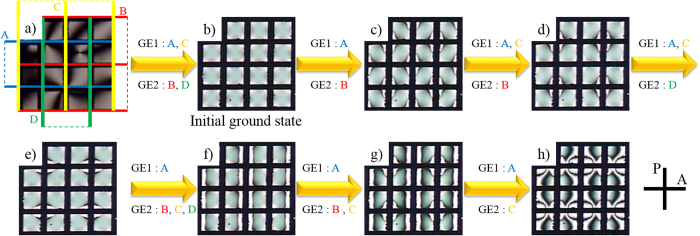
POM textures of the numerous ground states produced in a proposed LC cell. Each of the ground state is achieved by removing an electric field at *T* = 45 °C after *E* = 10 V/μm was applied on two groups of electrodes GE1 and GE2 (denoted above and under the arrows) at 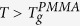. The electrodes A (blue line in Fig. 2a) and B (red line in Fig. 2a) are on a bottom substrate, and the electrodes C (yellow line in Fig. 2a) and D (green line in Fig. 2a) are on a top substrate. More possible ground states are shown in the Supplementary Information (Supplementary Figure S1).

Figure 2 shows the POM textures of numerous ground states resulting from the various combinations of *E* in a single LC cell. When the LC is injected into the cell, the cell initially exhibits a Schlieren texture as a ground state, Fig. 2 (a). In contrast to normal LC cells with a polyimide aligning layer, the proposed cell can transform its ground state from the Schlieren texture. For instance, the Schlieren texture (Fig. 2a) can be converted into the ground state shown in Fig. 2b as the alternating current (AC) *E* = 10 V/μm (sinusoidal wave of frequency *f* = 1 kHz) is introduced on the two groups of selected electrodes, GE1 (electrodes A and C) and GE2 (electrodes B and D), at 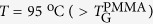 followed by removal of the field at *T* = 45 °C; the critical field E* to realign surface directors is smaller than 10 V/μm at *T* > 95 °C. With numerous combinations of electric fields, more than 20 different ground states could be derived from the initial ground state, Fig. 2b (see also Supplementary Figure S1 a1-a20). Each of the newly achieved ground state is stable as long as the cell is operating at *E* < E* and 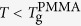.

**Figure 3:**
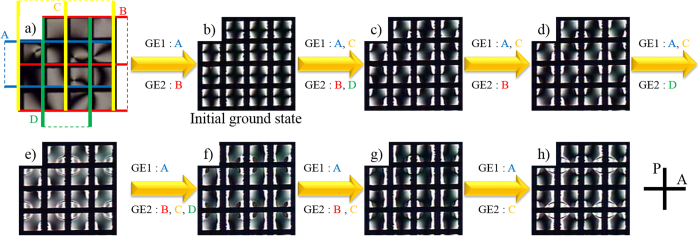
Another set of POM textures in the numerous ground states produced in a proposed LC cell. Each of the ground states is obtained in the same way as Fig. 2. Since the ex-ground states are different with the ones in Fig. 2, however, the resultant ground states are not identical as Fig. 2 although the same combination of fields is introduced. More possible ground states are shown in the Supplementary Information (Supplementary Figure S1).

Figure 3 shows another set of ground states derived from the initial ground state (Fig. 3b) that is different from Fig. 2b. The numerous ground states are obtained in the same way as Fig. 2. (see also Supplementary Figure S1 b1-b20).

From the results of Figs. 2 and 3, one finds that the reestablished ground states depend not only on the combinations of electrodes (GE1 and GE2) but also on the previous ground state. For instance, Fig. 2d and 3d are obtained by introducing *E* on the same combination of electrodes, electrodes A and C for GE1, and electrode B for GE2. However, Fig. 2d and 3d are not identical because their ex-ground states (Figs. 2c and 3c) are different. Therefore, a tremendous number of stable states are able to be generated by controlling both the ex-ground states and the combination of electrodes (See Supplementary Figure S1).

We experimentally verified that the newly established ground states are thermally and electrically stable for more than a day unless the cell is operated at *E* > E* and 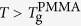. However, the procedure to reorientate the surface director 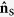 at a high temperature (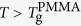) is still feasible to cause the side effects as follows. First, the PMMA layer in a glass phase at 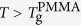 can be dissolved and diffused into LC molecules^25^,^26^, and thus cause impurities in the bulk of LC. The impurities would distort electro-optical properties and surface alignments of the cell^27^,^28^,^29^,^30^. Second, the operation of LC cells at a high temperature possibly results in a thermal deformation or degradation of aligning layer as well as LCs, leading to the corruption of characteristics in LC cells^7^,^30^,^31^,^32^. Therefore, in order to avoid the possible side effects, we theoretically described the surface gliding effect and examined the feasibility to realign 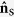 at a low temperature 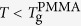 in our system.

When an in-plane electric field *E* is introduced in our system, the directors in the regions from *z* = 0 to *z* = *ξ*_*e*_ are twisted. The free energy *F* for the region can be expressed as
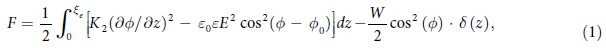
under the boundary conditions, 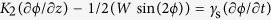 at *z* = 0 and *ϕ* = *ϕ*_*e*_ at *z* = *ξ*_*e*_^11^. Here, *K*_2_ is a twist elastic constant, *γ*_*s*_ is a surface viscosity, *φ* is the angle between a surface director and an easy axis, and *ϕ*_*e*_ is the angle between *E* and an easy axis. If it is assumed that the directors are twisted constantly at 0 < *z* < *ξ*_*e*_, one can obtain ∂*ϕ*/∂*z* = *ϕ*_*e*_/*ξ*_*e*_. Accordingly, the dynamics of 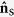 can be expressed as
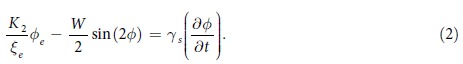
The solution of Eq. (2) is obtained by considering the small deviation angles *ϕ* as follows:
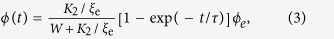
where *ξ*_*e*_ = 1/*E*(4*πK*_2_/Δ*ε*)^1/2^ and the time constant *τ* = γ_s_/(*W* + *K*_2_/*ξ*_*e*_).

**Figure 4:**
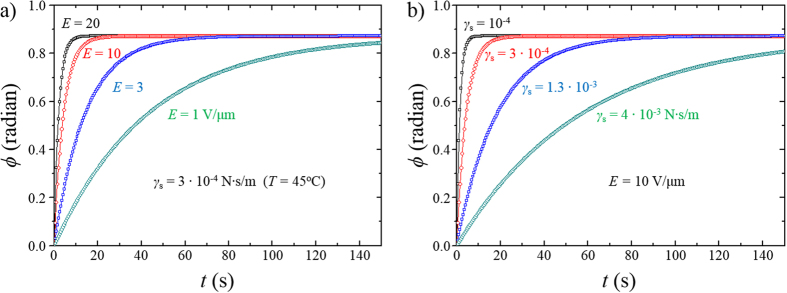
Theoretical estimation of the rotation angle *ϕ* of the surface director 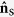 in the presence of an electric field. (**a**) Electric field *E* dependence of *ϕ* under the surface condition of PMMA layer at *T* = 45 °C; the surface viscosity *γ*_s_ of PMMA surface at *T* = 45 °C is *γ*_s_ = 3⋅10^−4^ N⋅s/m. (**b**) Surface viscosity dependence of *ϕ* in the presence of *E* = 10 V/μm under the surface conditions of PMMA layer at *T* ≤ 45 °C.

**Figure 5:**
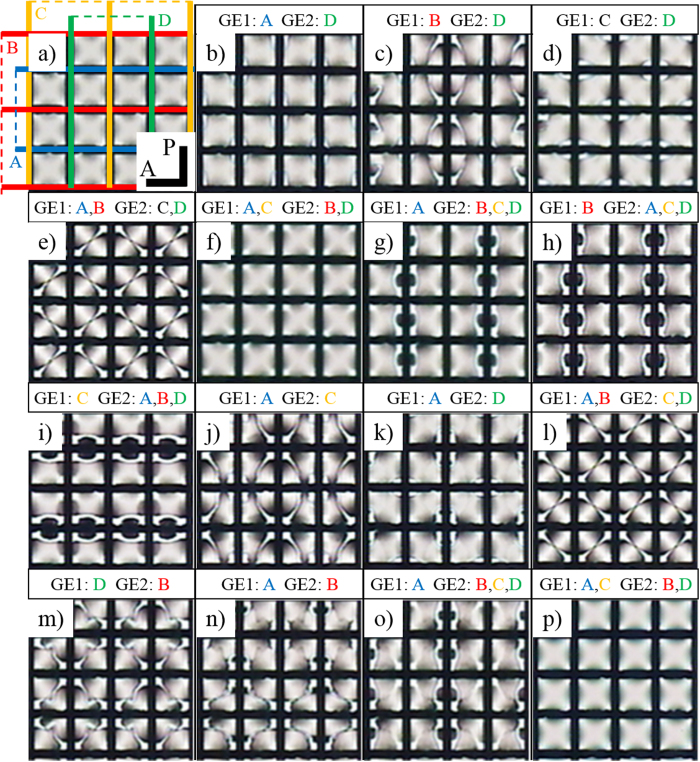
Alteration of ground states at *T* = 25 °C. Each ground state is established at *T* = 25 °C by removing the field after *E* = 15 V/μm was introduced on GE1 and GE2 (denoted on the top of each texture) for *t* = 30 s.

In our system, *ϕ*(*t* → ∞) = *ϕ*_*e*_ and *τ* = *γ*_s_*ξ*_*e*_/*K*_2_ because the surface anchoring *W* of PMMA layer (*W*_PMMA_ ≈ 10^−7^−10^−8^ N/m) is much smaller than *K*_2_/*ξ*_*e*_ ≈ 2.5 × 10^−5^ N/m. Therefore, if a strong *E* (>E*) is applied for a sufficient time *t* (>t*), 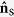 on the PMMA layer can be reoriented even at 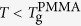. With Eq. (3), the azimuthal rotating angle *ϕ* of 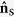 with *t* is estimated as a function of *E* (Fig. 4a) and *γ*_s_ (Fig. 4b). Figure 4a shows that even under the surface condition of PMMA layer at *T* = 45 °C (*γ*_s_ = 3⋅10^−4^ N⋅s/m), 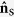 can be reorientated. For instance, the theoretical plot indicates that 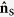 can be fully realigned at *T* = 45 ^o^C when *E* = 10 V/μm is applied for *t* > 15 s (red curve in Fig. 4a). Indeed, we could experimentally re-enact the alteration of ground states in Fig. 2 at *T* = 45 °C by applying *E* = 10 V/μm for *t* = 30 s. Figure 4b shows the *γ*_s_ dependence of the rotation of 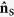 when *E* = 10 V/μm is introduced. The theoretical plot (blue and green curves in Fig. 4b) clearly indicates that 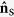 can be modified even at *T* < 45 °C; *γ*_*s*_ = 1.3⋅10^−3^ N⋅s/m (blue curve) correspond to the surface viscosity of PMMA at *T* = 25 °C. The transformation of ground states at *T* = 25 °C was experimentally demonstrated as shown in Fig. 5.

Figure 5 shows the various ground states obtained in a single LC cell by introducing numerous set of electric fields at *T* = 25 °C. As the theoretical examination predicted, we could indeed reorientate 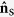 even at *T* = 25 °C by applying *E* = 15 V/μm for *t* = 30 s and thus realize numerous ground states after the field is removed. In this case, the resultant ground states do not depend on the previous ground states. For example, the same ground state (Fig. 5f,p) are derived from two different ground states (Fig. 5e,o) as *E* is applied on the same combination of electrodes (electrodes A and C for GE1, and electrode B and D for GE2). Any ground states can be restored by introducing the set of electric fields corresponding to the desired ground state. The re-established ground states are thermally and electrically stable as long as the cell is operated at 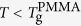 and *E* < E*.

## Discussion

In this study, we proposed a simple and novel configuration of LC cell to readily generate the multi-stable director alignments as its ground states using a surface gliding effect of nematic liquid crystal on a weak anchoring aligning surface. Thanks to the weak surface anchoring *W* and surface viscosity *γ*_s_ of the PMMA aligning layer, when a sufficient electric field *E* is applied, one can reorientate the director 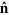 not only in a bulk but also in surface regions. Therefore, the realigned surface director 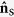 establish a new ground state when the field is removed. By adopting the interdigitated electrodes on both top and bottom substrates of the cell, various combinations of *E* can be produced, thereby generating the numerous ground states in a single cell. In addition, the dynamics of 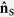 were examined theoretically. The theoretical estimation predicted that in our system 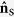 can be modified even at a room temperature if a sufficient *E* > E* and enough time *t* > t* are allowed in the system. In the proposed cell, we could indeed alter the ground states even at *T* = 25 °C and establish numerous ground states. The reestablished ground states are stable thermally and electrically unless the cell is operated at *E* > E* and 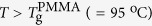.

As the market demand on the displaying device for static images (e. g., price tags, advertising board, electronic books, and menu) dramatically increases recently, the LCDs which can be of multi-stable ground states have been receiving renewed attention due to their ability for low power consumption. The simplicity and stability of the proposed system suggest that it can be a best candidate as the displaying devices for static images with a low power consumption and will find applications in various fields.

## Additional Information

**How to cite this article**: Gwag, J. S. *et al* Realization of Multi-Stable Ground States in a Nematic Liquid Crystal by Surface and Electric Field Modification. *Sci. Rep* **5**, 11368; doi: 10.1038/srep11368 (2015).

## Supplementary Material

Supplementary Information
