# Supplementary material for: Realization of Multi-Stable Ground States in a Nematic Liquid Crystal by Surface and Electric Field Modification
Source: Sci Rep. 2015 Jun 23;5:11368. doi: 10.1038/srep11368 (PMC4477365; doi:10.1038/srep11368)
Supplement: Supplementary Information [file srep11368-s1.pdf]

Supplementary Information

Realization of Multi-Stable Ground States in a Nematic Liquid Crystal by Surface and Electric Field Modification

Jin Seog Gwag[1], Young-Ki Kim[2], Chang Hoon Lee[3], and Jea-Hoon Kim[4,*]

[1]Department of Physics, Yeungnam University, Gyeongsan 712-749, Korea

[2]Liquid Crystal Institute and Chemical Physics Interdisciplinary Program, Kent State University, OH 44242

[3]Samsung Electronics, LCD R&D Center, Yongin 446-811, Korea

[4]Department of Electronics and Computer Engineering, Hanyang University, Seoul 133-791, Korea.

* jhoon@hanyang.ac.kr

Supplementary Figures

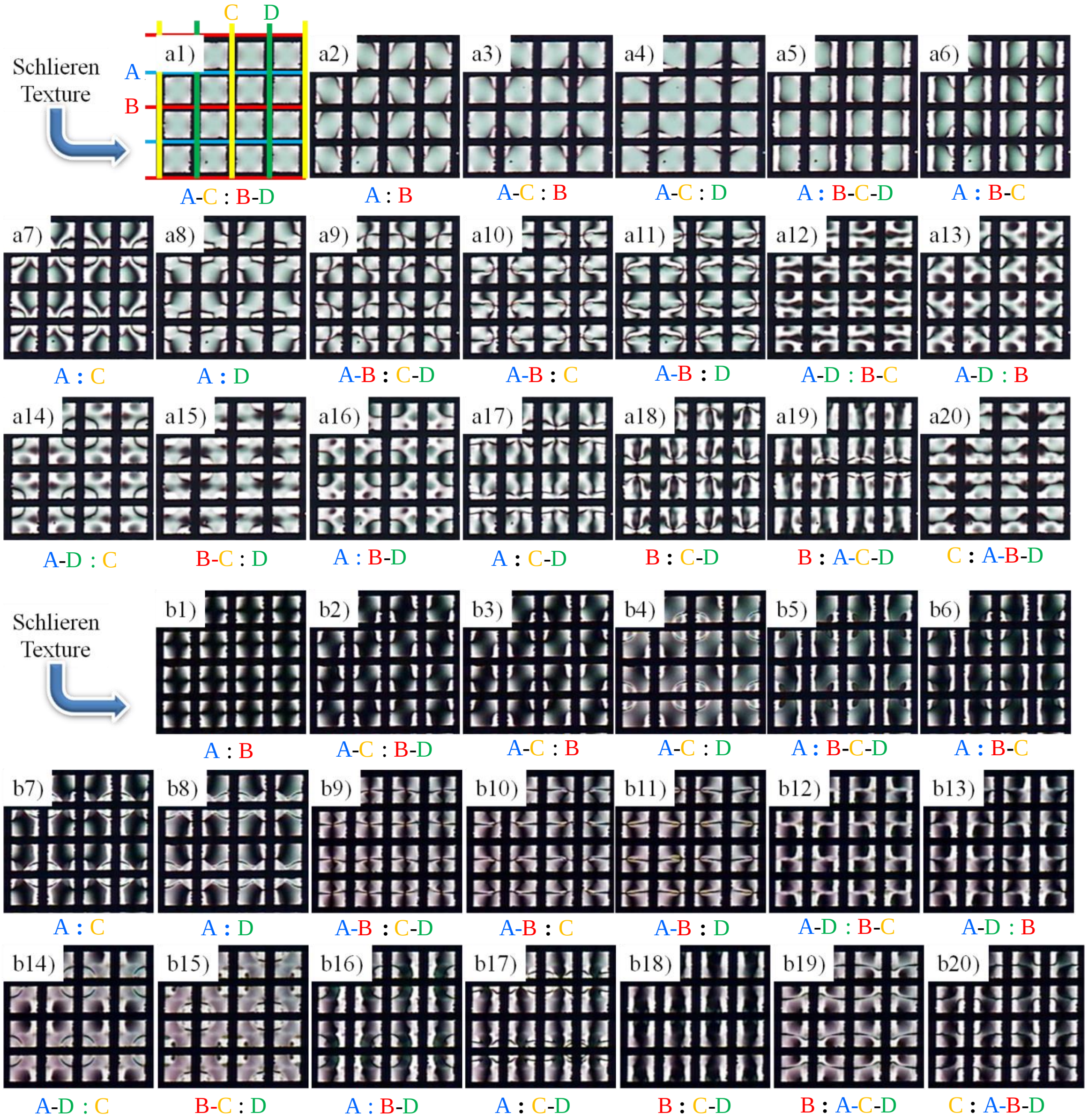

Supplementary Figure S1. Sequences of POM textures in ground states derived from two different initial states, a1 and b1. Each ground state is achieved as $E = 10\ \mathrm{V_{rms}}/\mu\mathrm{m}$ (Sinusoidal wave and frequency $f = 10\ \mathrm{Khz}$) was applied at $T > T_g^{\mathrm{PMMA}}$ on two groups of selected electrodes (denoted in the bottom of each texture) and subsequently the field was removed at $T = 45\,^{\circ}\mathrm{C}$.
